# The effect of transcutaneous electrical acupoint stimulation on postoperative awakening after general anaesthesia: a systematic review and meta-analysis

**DOI:** 10.3389/fmed.2024.1347641

**Published:** 2024-09-23

**Authors:** Shangkun Si, Xiaohu Zhao, Yuejun Mu, Li Xu, Fulei Wang, Dongbin Zhang, Fan Su

**Affiliations:** ^1^First Teaching Hospital of Tianjin University of Traditional Chinese Medicine, Tianjin, China; ^2^National Clinical Research Center for Chinese Medicine Acupuncture and Moxibustion, Tianjin, China; ^3^Tianjin University of Traditional Chinese Medicine, Tianjin, China; ^4^Shandong University of Traditional Chinese Medicine, Jinan, China; ^5^Yantai Hospital of Traditional Chinese Medicine, Yantai, China; ^6^Affiliated Hospital of Shandong University of Traditional Chinese Medicine, Jinan, China

**Keywords:** acupuncture points, anaesthesia recovery period, general anaesthesia, meta-analysis, perioperative period, surgery, systematic review, transcutaneous electric nerve stimulation

## Abstract

**Background:**

The existing body of research concerning the impact of transcutaneous electrical acupoint stimulation (TEAS) on early postoperative recovery is marked by a lack of consensus. This meta-analysis, encompassing a systematic review of randomised controlled trials, seeks to critically assess the efficacy of TEAS in relation to awakening from general anaesthesia in the postoperative period.

**Methods:**

The inclusion criteria for this study were peer-reviewed randomised controlled trials that evaluated the influence of TEAS on the process of regaining consciousness following general anaesthesia. A comprehensive search was conducted across several reputable databases, including PubMed, Embase, the Cochrane Library, the China National Knowledge Infrastructure, the VIP Database, the SinoMed Database, and the WANFANG Medical Database. The search was not limited by date, extending from the inception of each database up to December 2023. The methodological quality and risk of bias within the included studies were appraised in accordance with the guidelines outlined in the Cochrane Handbook for Systematic Reviews of Interventions, version 5.1, and its associated tool for assessing risk of bias.

**Results:**

The analysis encompassed 29 studies involving a total of 2,125 patients. Participants in the TEAS group demonstrated a significantly shorter duration to achieve eye-opening [mean difference (MD), −3.16 min; 95% confidence interval (CI), −3.93 to −2.39], endotracheal extubation (MD, −4.28 min; 95% CI, −4.79 to −3.76), and discharge from the post-anaesthesia care unit (MD, −8.04 min; 95% CI, −9.48 to −6.61) when compared to the control group receiving no or sham stimulation. Additionally, the TEAS group exhibited markedly reduced mean arterial blood pressure (MD, −9.00 mmHg; 95% CI, −10.69 to −7.32), heart rate (MD, −7.62 beats/min; 95% CI, −9.02 to −6.22), and plasma concentrations of epinephrine (standardised MD, −0.81; 95% CI, −1.04 to −0.58), norepinephrine (MD, −47.67 pg/ml; 95% CI, −62.88 to −32.46), and cortisol (MD, −110.92 nmol/L; 95% CI, −131.28 to −90.56) at the time of extubation. Furthermore, the incidence of adverse effects, including agitation and coughing, was considerably lower in the TEAS group relative to the control group (odds ratio, 0.30; 95% CI, 0.22–0.40).

**Conclusion:**

The findings of this study indicate that TEAS may hold promise in facilitating the return of consciousness, reducing the interval to awakening post-general anaesthesia, and enhancing the awakening process to be more tranquil and secure with a diminished likelihood of adverse events. However, caution must be exercised in interpreting these results due to the notable publication and geographical biases present among the studies under review. There is an imperative for further high-quality, low-bias research to substantiate these observations.

**Systematic review registration:**

The review protocol was registered with the PROSPERO International Prospective Register of Systematic Reviews (CRD42022382017).

## 1 Introduction

Prolonged awakening following general anaesthesia and adverse reactions during the awakening phase, such as stress disorders, hemodynamic instability, and agitation, can significantly compromise the quality of postoperative recovery (1). Narcotic antagonists and sedative-analgesic medications are commonly employed in the treatment of delayed awakening and agitation; however, they present risks including respiratory depression, nausea, vomiting, and potentially adverse cardiovascular events (2).

Acupuncture and acupoint stimulation, as non-pharmacological, complementary, and alternative therapies, offer a safe and efficacious approach with minimal side effects. They have been proposed to play a beneficial role in perioperative medicine, where they serve to reduce the dosage of anaesthetic agents, mitigate stress responses, protect organ function, decrease the incidence of complications, and enhance the quality of postoperative recovery, as supported by an extensive body of research (3). Transcutaneous electrical acupoint stimulation (TEAS) represents a non-invasive electrical stimulation modality, integrating transcutaneous electrical nerve stimulation from Western medicine with acupoint stimulation from traditional Chinese medicine. TEAS is typically administered by affixing self-adhesive electrodes, connected to a transcutaneous electrical stimulator, to the patient’s acupoints, with the stimulator parameters adjusted to provide the desired stimulation. It shares comparable therapeutic effects with traditional acupuncture, yet offers the advantages of being non-invasive and non-contagious, leading to high patient acceptance and practicality (4).

The current evidence regarding the efficacy of TEAS in the context of early postoperative recovery is inconsistent (4–7). Furthermore, there is a notable absence of meta-analyses specifically addressing the impact of TEAS on awakening following general anaesthesia. Consequently, informed by the principles of evidence-based medicine, this meta-analysis was conducted to evaluate the impact of TEAS on postoperative awakening after general anaesthesia, with the aim of contributing to the existing body of evidence.

## 2 Materials and methods

### 2.1 Protocol and registration

This study adheres to the Preferred Reporting Items for Systematic Reviews and Meta-Analyses (PRISMA) guidelines and the Assessing the Methodological Quality of Systematic Reviews (AMSTAR) guidelines (8, 9). The PRISMA checklist is provided in Supplementary Table 1. The study design follows the recommendations of the Cochrane Handbook for Systematic Reviews of Interventions. The review protocol was registered with the PROSPERO International Prospective Register of Systematic Reviews (CRD42022382017).

### 2.2 Search strategy

The databases searched included PubMed, Embase, the Cochrane Library, the China National Knowledge Infrastructure, the VIP Database, the SinoMed Database, and WANFANG Medical. The search spanned from the inception of each database to December 2023, without restrictions on language or publication date. The search strategy encompassed free-text terms such as: “general anaesthesia,” “awakening,” “emergence,” “recovery,” “open eyes,” “extubation,” “PACU (Post-anaesthesia Care Unit),” “acupoint,” “acupuncture,” “TAES,” and “electroacupuncture.”

Variations of these terms were also included, and the reference lists of relevant articles were manually reviewed for potentially eligible studies. The complete search strategy is detailed in Supplementary Table 2.

### 2.3 Eligibility criteria

Inclusion criteria were as follows: (1) Study type: randomised controlled trials (RCTs). (2) Study subjects: patients undergoing general anaesthesia and surgery who received TEAS or blank/sham stimulation during the perioperative period, without restrictions on age, gender, or nationality. (3) Intervention: the TEAS group received TEAS during the perioperative period; the blank/sham stimulation group received no acupuncture stimulation or received stimulation at non-meridian, non-acupoint locations.

Exclusion criteria included: (1) Study type: prospective cohort studies/retrospective case-control studies/non-randomised studies, comments, editorials, letters, case reports, reviews, conference proceedings, or animal studies. (2) Study subjects/intervention: non-general anaesthesia surgical patients or other acupuncture therapy-related interventions (e.g., manual acupuncture, electroacupuncture, ear point pressing beans, acupoint catgut embedding, acupressure, acupoint injection, etc.) were included in the study. (3) Studies where the original text was not available or where outcome indicators were incomplete.

### 2.4 Outcome indicators

The primary outcome measure of this study was the quality of awakening, which includes the speed of awakening (primary outcomes) and the smoothness of awakening (secondary outcomes). The speed of awakening encompassed the time to open eyes (minutes), time to extubation (minutes), and time to leave the PACU (minutes). The smoothness of awakening included hemodynamic stability at extubation, plasma stress hormone levels at extubation, and adverse reactions during the awakening period. Hemodynamic stability was assessed by mean arterial pressure (MAP, mmHg) and heart rate (HR, beats/min). Plasma stress hormone levels included those of epinephrine (E, standardised mean difference, units not specified), norepinephrine (NE, pg/ml), and cortisol (Cor, nmol/L). Adverse reactions during the awakening period encompassed agitation and cough.

### 2.5 Data extraction

Note Express v3.5.0 was utilised for managing the included research literature. Office Excel was employed for creating tables and summarising, deduplicating, screening, and extracting research data from the literature. The literature was initially screened by title and abstract, followed by a secondary screening based on the full text. Data such as authors’ names, years of publication, sample size, interventions, and outcome indicators were extracted from the final publications of the studies. Two researchers (authors of this work) performed data extraction separately and independently, followed by cross-checking. In the event of discrepancies, the matter was referred to the corresponding author for arbitration.

### 2.6 Quality assessment

The quality of the included studies was evaluated according to the Cochrane Systematic Review Manual 5.1 and its recommended Risk of Bias Assessment Tool. The studies were assessed for “random sequence generation,” “allocation concealment,” “blinding of participants and trial personnel,” “blinding of outcome assessors,” “incomplete outcome data,” “selective reporting,” and “other biases.” The results of the bias assessment were categorised as “low risk,” “high risk,” or “unclear.” The quality assessment was conducted separately and independently by two researchers (authors of this work) and then cross-checked. Disagreements were resolved by referring to the corresponding author for arbitration.

### 2.7 Statistical analysis

Statistical analysis was conducted using Review Manager (RevMan, version 5.3, Cochrane Collaboration). Dichotomous variables were analysed using the odds ratio (OR) or relative risk (RR) with a 95% confidence interval (CI). Continuous variables were assessed with the mean difference (MD) or standardised mean difference (SMD) and 95% CI. Inter-study heterogeneity was evaluated using a Chi-squared test at a significance level of α = 0.1, with the degree of heterogeneity determined based on *I*^2^ values. A *P*-value ≥ 0.1 and *I*^2^ value ≤50% indicated good homogeneity among the included studies, prompting the use of a fixed-effects model and OR for meta-analysis. A *P*-value < 0.1 and *I*^2^ value >50% indicated significant heterogeneity, necessitating subgroup analysis or sensitivity analysis to identify the source. If no significant clinical heterogeneity was present or the results were stable, the random-effects model and RR were selected for meta-analysis. Sensitivity analysis was performed to assess the stability of the results when studies with large weights were included. If the results from studies with substantial heterogeneity could not be reasonably addressed following these approaches (e.g., subgroup analysis and sensitivity analysis), meta-analysis was not conducted, and only descriptive statistics were provided. If the number of included studies was sufficient (more than 10), the risk of publication bias was evaluated using funnel plots (visually) and the Egger regression test (Stata, version 17.0, Stata Corp., College Station, TX, USA), with a *P*-value < 0.1 considered indicative of significant publication bias (10).

## 3 Results

### 3.1 Literature search

**FIGURE 1:**
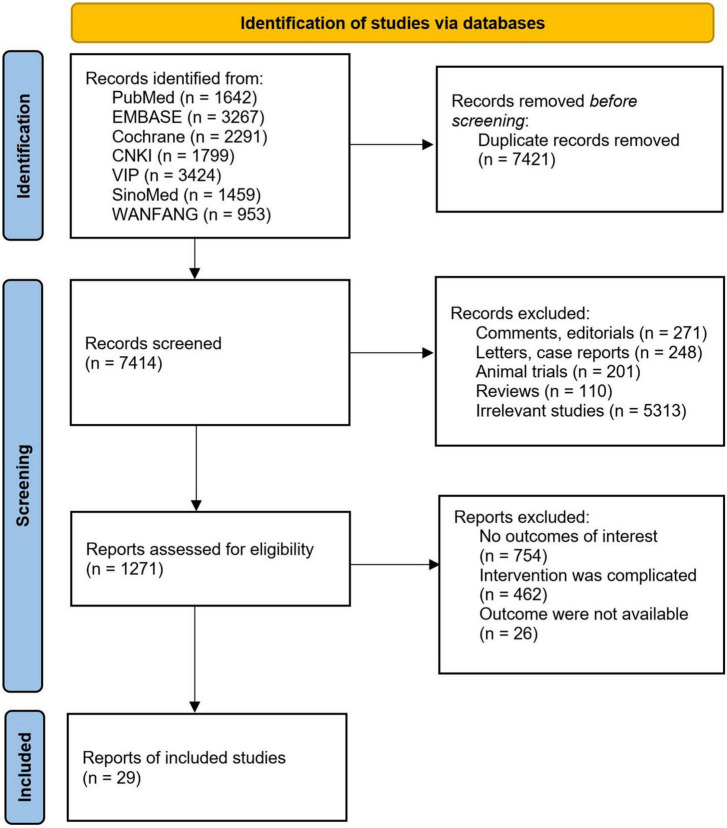
PRISMA (preferred reporting items for systematic reviews and meta-analyses) flow diagram.

The initial search yielded 14,835 potentially relevant citations. After the exclusion of 7,421 duplicates, 6,143 based on title and abstract, and 1,242 based on full-text assessment, 29 studies meeting the inclusion criteria were ultimately included (11–38), encompassing a total of 2,125 patients, with 1,060 in the TEAS group and 1,065 in the blank/sham stimulation group. The literature screening process is depicted in Figure 1. The basic characteristics of the included studies are outlined in Tables 1, 2.

**TABLE 1:** Characteristic of the included studies.

References	Country	Surgery	Anaesthesia	Patients	Sample size		Outcome
					**T**	**C**	
Li et al. (11)	China	OS	IIA	Children	29	29	② ⑨
Meng et al. (12)	China	TS	TIVA	Adult	40	40	⑨
Xing et al. (13)	China	LGGC	TIVA+NB	The aged	29	29	② ④ ⑤
Gao et al. (14)	China	UL	TIVA	AM	29	28	① ③
Jin et al. (15)	China	RMBC	TIVA	AF	30	31	②
Zhan et al. (16)	China	LIHR	TIVA	Children	40	40	① ③ ⑨
Zhang et al. (17)	China	TP	TIVA	Adult	42	42	④ ⑤ ⑥ ⑦ ⑧
Nakamura et al. (18)	Japan	IRTF	IIA+NB	Children	50	50	⑨
Yang et al. (19)	China	RGS	IIA	AF	47	47	① ②
Bai et al. (20)	China	SC	IIA	The aged	37	38	② ④ ⑤ ⑥ ⑦ ⑧ ⑨
Bai et al. (21)	China	THY	IIA	Adult	30	30	④ ⑤ ⑥ ⑦ ⑧
Huang et al. (22)	China	LOL	IIA	Adult	20	20	② ③
Lin et al. (23)	China	GS	IIA	AF	70	70	① ②
Bai et al. (24)	China	CRA	TIVA	The aged	30	30	④ ⑤ ⑥ ⑦ ⑨
Guo et al. (25)	China	THY	IIA	Adult	30	30	① ② ④ ⑤ ⑨
Hijikata et al. (26)	Japan	MT	IIA+NB	Children	60	60	⑨
Zhu et al. (27)	China	GLS	IIA	AF	30	30	② ④ ⑤ ⑥ ⑦ ⑧ ⑨
Chen et al. (28)	China	THY	IIA	AF	41	42	③
Liu et al. (29)	China	SC	TIVA	Adult	44	44	① ② ③
Yao et al. (30)	China	GLS	IIA	AF	35	36	③
Wang et al. (31)	China	SIN	TIVA	Adult	30	30	②
Chen et al. (32)	China	SC	IIA	Adult	40	40	① ② ③ ⑨
Gong et al. (33)	China	OS	TIVA	The aged	40	40	① ② ④ ⑤ ⑨
Xing et al. (34)	China	THY	TIVA	Unknown	30	30	② ③ ④ ⑤ ⑥ ⑦
Yang et al. (35)	China	GLS	IIA	AF	30	30	① ② ④ ⑤
Yu et al. (36)	China	RMBC	TIVA	AF	30	30	② ⑨
Yang et al. (37)	China	GLS	IIA	AF	30	30	① ② ⑤ ⑨
Coloma et al. (38)	USA	LS	IIA	Adult	30	30	③
White et al. (39)	USA	SPS	IIA	Adult	37	39	③

T, TEAS group; C, controlled group; IIA, inhalational-intravenous anaesthesia; TIVA, total intravenous anaesthesia; NB, nerve block; OS, orthopedic surgery; TS, thoracoscopic surgery; LGGC, laparoscopic gastrectomy for gastric cancer; UL, ureteroscopic lithotripsy; RMBC, radical mastectomy for breast cancer; LIHR, laparoscopic inguinal hernia repair; TP, thoracoscopic pneumonectomy; IRTF, inguinal repair/testicular fixation surgery; RGS, robotic gynecologic surgery; SC, supratentorial craniotomy; THY, thyroidectomy; LOL, lobectomy of lung; GS, gynecologic surgery; CRA, craniotomy; MT, multiple types; GLS, gynecological laparoscopic surgery; SIN, sinusotomy; OS, open surgery; LS, laparoscopic surgery; SPS, surgical plastic surgery; AM, adult males; AF, adult females; ①, time to open eyes; ②, time to extubation; ③, time to leave the PACU; ④, MAP (immediately after extubation); ⑤, HR (immediately after extubation); ⑥, E (immediately after extubation); ⑦, NE (immediately after extubation); ⑧, Cor (immediately after extubation); ⑨, adverse reactions.

**TABLE 2:** Details of interventions.

References	Device	Time point	Frequency, current	Acupoint
Li et al. (11)	SDZ-V ENTI	10 min before the induction till the end of surgery	2/10 Hz, 10∼15 mA	bil (LI4, PC6)
Meng et al. (12)	SDZ-II ENTI	30 min before the induction till the end of surgery	2/100 Hz, 3∼8 mA	bil (LI4, ST36, SP6)
Xing et al. (13)	SDZ-V ENTI	30 min before the induction till the end of surgery	2/100 Hz, unknown	bil (LI4, PC6, ST36)
Gao et al. (14)	SDZ-V ENTI	Before induction, and lasted for 30 min	2/15 Hz, 6∼10 mA	uk (RN4, RN3, ST36, SP6)
Jin et al. (15)	HANS-200A	30 min before the induction till the end of surgery	2/100 Hz, 6∼12 mA	bil (LI4, PC6, ST36, SP6)
Zhan et al. (16)	HANS-200E	30 min before the induction till the end of surgery	2/100 Hz, 6∼10 mA	bil (LI4, PC6)
Zhang et al. (17)	HANS-200	30 min before the induction till the end of surgery	2/100 Hz, 8∼15 mA	bil (PC6, LI4, LU7, LU5)
Nakamura et al. (18)	NTM	After induction till the end of surgery	1 Hz, 50 mA	unil (HT7)
Yang et al. (19)	ENTI	30 min before the induction till the end of surgery	2/10 Hz, unknown	uk (ST36, SP6, BL60, BL59)
Bai et al. (20)	SDZ-II ENTI	30 min before the induction till 5 min before the end of surgery	2/10 Hz, 6∼15 mA	unil (LI4, PC6, LU7, LU5, LI18, ST9)
Bai et al. (21)	SD-II ENTI	30 min before the induction till 5 min before the end of surgery	2/100 Hz, 6∼15 mA	bil (LI4, PC6, LU7, LU5)
Huang et al. (22)	HANS-200A	Before induction and during surgery, lasted for 30 min	2/100 Hz, unknown	unil (PC6, LI4, LU7, LI11)
Lin et al. (23)	HANS-LH-202	After induction till the end of surgery	2/100 Hz, 8∼15 mA	bil (ST36, SP6)
Bai et al. (24)	SDZ-II ENTI	30 min before the induction till 5 min before the end of surgery	2/100 Hz, 8∼12 mA	uk (LI4, PC6, ST36)
Guo et al. (25)	HANS-200A	30 min before the induction till the end of surgery	2/10 Hz, unknown	bil (LI18, LI4, PC6)
Hijikata et al. (26)	PNS	Until the end of the operation	1 Hz, 50 mA	bil (HT7)
Zhu et al. (27)	SD-II ENTI	30 min before induction till 5 min before the end of surgery	2/100 Hz, unknown	bil (LI4, PC6, LU7, LU5, LI18)
Chen et al. (28)	HANS-100A	Before the induction, and lasted for 30 min	2/10 Hz, 6∼9 mA	bil (LI4, PC6)
Liu et al. (29)	HANS-LH-202H	30 min before the induction till the end of surgery	2/100 Hz, unknown	unil (LI4, TE5, BL63, LR3, ST36, GB40, GB20, BL10, BL2, EX-HN4)
Yao et al. (30)	HANS-100B	Before the induction, and lasted for 30 min	2/10 Hz, 6∼9 mA	bil (LI4, PC6, ST36, SP6)
Wang et al. (31)	SDZ-V ENTI	Before the induction, and lasted for 30 min	2/10 Hz, 6∼9 mA	bil (LI4, PC6, ST36)
Chen et al. (32)	HANS-LH-202H	Before the induction till the end of surgery	2/100 Hz, 8∼12 mA	unil (LI4, SJ5, BL63, LR3, ST36, GB40)
Gong et al. (33)	G6805-2 ENTI	20 min before the induction	2/20 Hz, unknown	uk (PC6, LI4, ST36)
Xing et al. (34)	HANS	20 min before the induction till extubation	2/100 Hz, 8∼12 mA	bil (PC6, LI4)
Yang et al. (35)	HANS-LH-202H	20∼30 min before the induction till the end of surgery	2/100 Hz, 5∼15 mA	bil (LI4, LR3)
Yu et al. (36)	HANS-LH-402	30 min before induction till the end of surgery	2/100 Hz, 5∼10 mA	unil (LI4, PC8, PC6, SJ5)
Yang et al. (37)	HANS-LH-202H	20∼30 min before the induction till the end of surgery	2/100 Hz, 12∼15 mA	bil (LI4, LR3)
Coloma et al. (38)	ReliefBand	In PACU	10∼35 mA	unil (PC6)
White et al. (39)	ReliefBand	In PACU	Unknown	unil (PC6)

HANS, Han’s acupoint nerve stimulator; ENTI, electrodes piece connected to the electronic needle therapy instrument; NTM, neuromuscular transmission monitoring devices; PNS, peripheral nerve stimulator; bil, bilateral; unil, unilateral; uk, unknown (it was not clear whether it was unilateral or bilateral); BL, BLadder; EX-HN, EXtra Head and Neck; GB, Gall Bladder; HT, HearT; LI, Large Inte Stine; LU, LUng; LR, LiveR; SJ, SanJiao (Chinese phonetic alphabet, also known as TE, Triple Energizer); ST, STomach; P(PC), Peri Cardium; RN, ReN (Chinese phonetic alphabet, conception vessel). The Chinese names and location of acupoints in the table can be viewed in Supplementary Table 3.

### 3.2 Quality assessment of included studies

**FIGURE 2:**
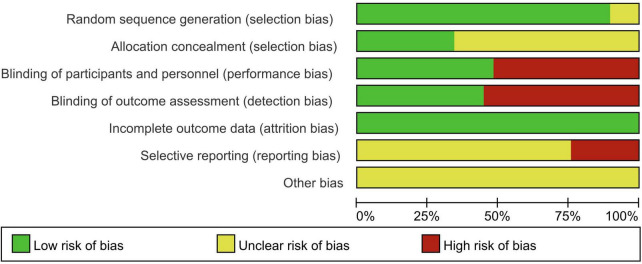
Percentage plot of the risk of bias of the included studies.

**FIGURE 3:**
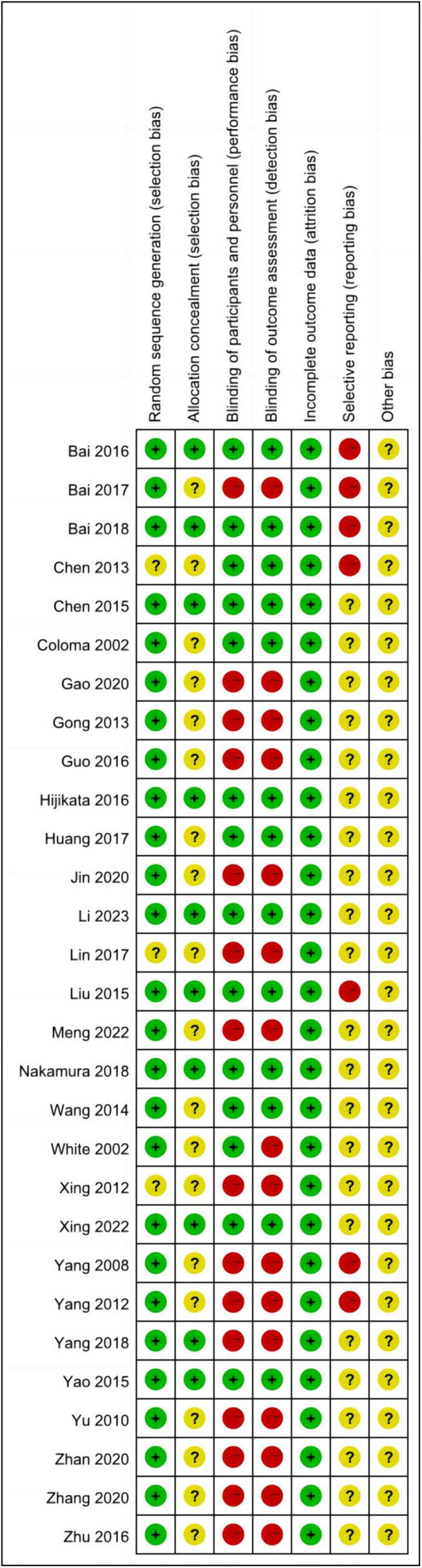
Summary of the risk of bias of the included studies.

The quality of the studies was evaluated using the Cochrane Risk of Bias Assessment Tool, and the overall quality was deemed satisfactory (Figures 2, 3). The certainty of evidence (GRADE) for each outcome is summarised in Table 3. Specific findings are as follows:

**TABLE 3:** Certainty of evidence (grade).

Outcome (participants/studies)	Risk of bias	Inconsistency	Indirectness	Imprecision	Overall certainty
Time to open eyes 719 (9 RCTs)	Serious	Not serious	Not serious	Serious	⊕⊕○○ Low
Time to extubation 1,001 (15 RCTs)	Not serious	Not serious	Not serious	Not serious	⊕⊕⊕⊕ High
Time to leave the PACU 635 (9 RCTs)	Not serious	Not serious	Not serious	Serious	⊕⊕⊕○ Moderate
MAP at extubation 657 (10 RCTs)	Serious	Not serious	Not serious	Not serious	⊕⊕⊕○ Moderate
HR at extubation 637 (10 RCTs)	Serious	Not serious	Not serious	Not serious	⊕⊕⊕○ Moderate
Epinephrine at extubation 315 (5 RCTs)	Serious	Not serious	Not serious	Not serious	⊕⊕⊕○ Moderate
Norepinephrine at extubation 399 (6 RCTs)	Serious	Not serious	Not serious	Not serious	⊕⊕⊕○ Moderate
Cortisol at extubation 204 (3 RCTs)	Serious	Not serious	Not serious	Not serious	⊕⊕⊕○ Moderate
Adverse reactions during the awakening period 1,110 (9 RCTs)	Serious	Not serious	Not serious	Serious	⊕⊕○○ Low

1. Random sequence generation: Thirteen studies (11, 12, 15–17, 19, 21, 25, 30, 33, 35–37) utilised the random number table method. An additional 11 studies (13, 14, 18, 20, 22, 26, 28, 29, 31, 38, 39) employed computer-generated random grouping. One study employed envelope random assignment, another used stratified randomisation. Two studies (23, 32) referenced randomisation without detailing the sequence generation method, and one study (34) made no mention of randomisation.
2. Allocation concealment: Ten studies (11, 13, 18–20, 24, 26, 28–30) detailed the use of closed-envelope allocation concealment; the remaining studies did not report on allocation concealment.
3. Blinding of subjects and trial personnel: Thirteen studies (11, 13, 18, 20, 22, 26, 28–32, 38, 39) reported the implementation of double-blinding. One study (24) blinded study personnel, while the remainder did not specify blinding methods.
4. Blinding of outcome assessors: Thirteen studies (11, 13, 18, 20, 22, 24, 26, 28–32, 38) reported the blinding of outcome assessors. The remaining studies did not mention the blinding of outcome assessment.
5. Incomplete outcome data: Five studies (13, 15, 26, 28, 29) reported missing data, including withdrawals and lost visits, with no significant impact on effect sizes. The remaining studies reported no missing data.
6. Selective reporting: Of the 29 enrolled studies, 25 originated from China, which may introduce geographical bias. Four studies (20, 24, 29, 32) focused on craniotomies, potentially susceptible to publication bias. Three studies (20, 21, 24) were conducted by the team of Bai W, and two studies (35, 37) by the team of Yang Q, which could also contribute to publication bias.
7. Other bias: There was insufficient evidence or information to assess whether other serious risks of bias were present in the included studies.

### 3.3 The speed of awakening

**FIGURE 4:**
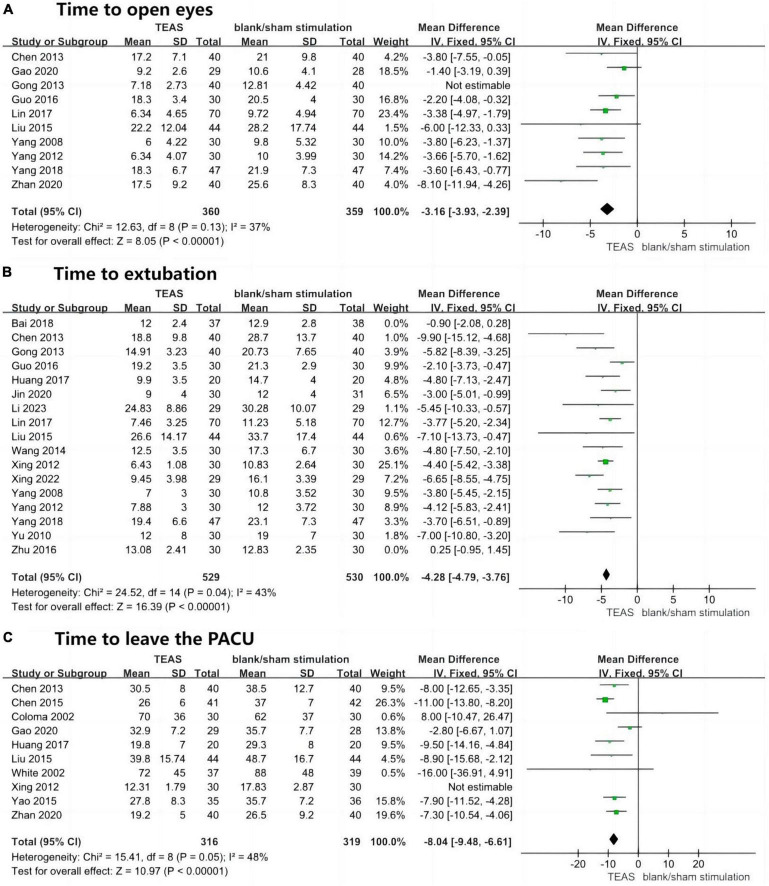
Forest plots (speed of awakening). **(A)** Time to open eyes (min). **(B)** Time to extubation (min). **(C)** Time to leave the PACU (min). TEAS, transcutaneous electrical acupoint stimulation group; control, blank/sham stimulation group.

Ten studies reported the time to eye opening in the TEAS group compared to the blank/sham stimulation group after general anaesthesia, involving 799 patients—400 in the TEAS group and 399 in the control group. The results indicated significant statistical heterogeneity (*P* = 0.02, *I*^2^ = 55%), leading to the exclusion of one study after sensitivity analysis (33). Upon retesting, heterogeneity was no longer significant (*P* = 0.13, *I*^2^ = 37%), with low sensitivity and good stability. A fixed-effects model was thus employed for effect size calculation and analysis. The aggregated results demonstrated that the TEAS group had a significantly shorter time to eye opening compared to the blank/sham stimulation group (MD, −3.16 min; 95% CI, −3.93 to −2.39 min; *P* < 0.001), as depicted in Figure 4A.

Seventeen studies reported the time to extubation in the TEAS group compared to the blank/sham stimulation group after general anaesthesia, involving 1,194 patients—596 in the TEAS group and 598 in the control group. Initial results showed significant statistical heterogeneity (*P* < 0.001, *I*^2^ = 82%), and after sensitivity analysis, two studies were excluded (20, 27). Retesting revealed no significant heterogeneity (*P* = 0.04, *I*^2^ = 43%), with low sensitivity and good stability. A fixed-effects model was used for effect size calculation and analysis. The overall results indicated that the TEAS group had a significantly shorter time to extubation compared to the blank/sham stimulation group (MD, −4.28 min; 95% CI, −4.79 to −3.76 min; *P* < 0.001), as shown in Figure 4B.

Ten studies reported the duration in the PACU in the TEAS group compared to the blank/sham stimulation group after general anaesthesia, involving 695 patients—346 in the TEAS group and 349 in the control group. Results showed significant statistical heterogeneity (*P* = 0.008, *I*^2^ = 60%), leading to the exclusion of one study after sensitivity analysis (34). Retesting showed no significant heterogeneity (*P* = 0.05, *I*^2^ = 48%), with low sensitivity and good stability. A fixed-effects model was used for effect size calculation and analysis. The overall results demonstrated that the TEAS group had a significantly shorter duration in the PACU compared to the blank/sham stimulation group (MD, −8.04 min; 95% CI, −9.48 to −6.61 min; *P* < 0.001), as shown in Figure 4C.

### 3.4 The smoothness of awakening

**FIGURE 5:**
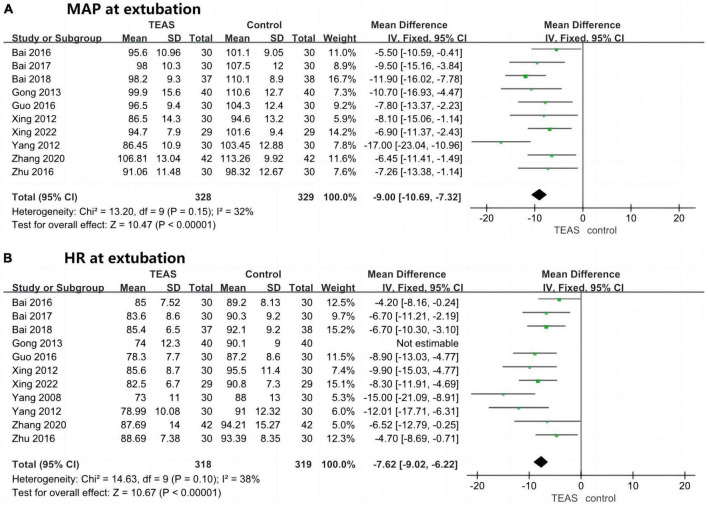
Forest plots (hemodynamic stability at extubation). **(A)** MAP at extubation (mmHg). **(B)** HR at extubation (beats/min). TEAS, transcutaneous electrical acupoint stimulation group; Control, blank/sham stimulation group.

Ten studies reported on MAP at extubation in the TEAS group compared to the blank/sham stimulation group, involving 657 patients—328 in the TEAS group and 329 in the control group. Results did not indicate significant statistical heterogeneity (*P* = 0.15, *I*^2^ = 32%), with low sensitivity and good stability. A fixed-effects model was used for effect size calculation and analysis. The overall results showed that the MAP at extubation was significantly lower in the TEAS group than in the blank/sham stimulation group (MD, −9.00 mmHg; 95% CI, −10.69 to −7.32 mmHg; *P* < 0.001), as depicted in Figure 5A.

Eleven studies reported on HR at extubation in the TEAS group compared to the blank/sham stimulation group, involving 717 patients—358 in the TEAS group and 359 in the control group. Initial results showed significant statistical heterogeneity (*P* = 0.004, *I*^2^ = 62%), and after sensitivity analysis, one study was excluded (33). Retesting revealed no significant heterogeneity (*P* = 0.10, *I*^2^ = 38%), with low sensitivity and good stability. The overall results indicated that the HR at extubation was significantly lower in the TEAS group than in the blank/sham stimulation group (MD, −7.62 beats/min; 95% CI, −9.02 to −6.22 beats/min; *P* < 0.001), as shown in Figure 5B.

**FIGURE 6:**
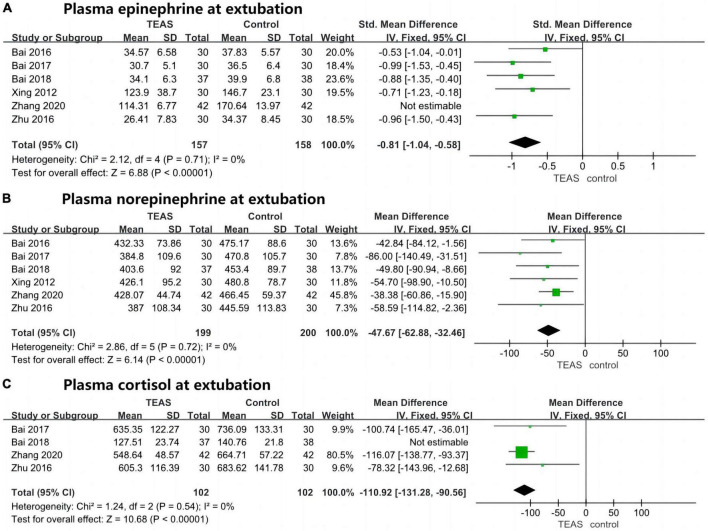
Forest plots (plasma stress hormone levels at extubation). **(A)** Plasma epinephrine at extubation (standardised mean difference, without units). **(B)** Plasma norepinephrine at extubation (pg/ml). **(C)** Plasma cortisol at extubation (nmol/L). TEAS, transcutaneous electrical acupoint stimulation group; control, blank/sham stimulation group.

Six studies reported on plasma epinephrine levels at extubation in the TEAS group compared to the blank/sham stimulation group, involving 399 patients—199 in the TEAS group and 200 in the control group. Initial results showed significant statistical heterogeneity (*P* < 0.001, *I*^2^ = 94%), leading to the exclusion of one study after sensitivity analysis (17). Retesting showed no significant heterogeneity (*P* = 0.71, *I*^2^ = 0%), with low sensitivity and good stability. The overall results demonstrated that plasma epinephrine levels at extubation were significantly lower in the TEAS group than in the blank/sham stimulation group (SMD, −0.81; 95% CI, −1.04 to −0.58; *P* < 0.001), as shown in Figure 6A.

Six studies compared the TEAS group with the blank/sham stimulation group regarding plasma norepinephrine levels at extubation, involving a total of 399 patients—199 in the TEAS group and 200 in the control group. The analysis did not reveal significant statistical heterogeneity (*P* = 0.72, *I*^2^ = 0%), indicating low sensitivity and good stability. A fixed-effects model was thus employed for effect size calculation and analysis. The combined results demonstrated that plasma norepinephrine levels at extubation were significantly lower in the TEAS group than in the blank/sham stimulation group (MD, −47.67 pg/ml; 95% CI, −62.88 to −32.46 pg/ml; *P* < 0.001), as illustrated in Figure 6B.

Four studies evaluated the levels of plasma cortisol at extubation between the TEAS group and the blank/sham stimulation group, including 279 patients—139 in the TEAS group and 140 in the control group. Initial analysis showed significant statistical heterogeneity (*P* < 0.001, *I*^2^ = 96%), prompting the exclusion of one study after sensitivity analysis (20). Subsequent analysis did not indicate significant heterogeneity (*P* = 0.54, *I*^2^ = 0%), with low sensitivity and good stability. A fixed-effects model was used for effect size calculation and analysis. The results showed that plasma cortisol levels at extubation were significantly lower in the TEAS group compared to the blank/sham stimulation group (MD, −110.92 nmol/L; 95% CI, −131.28 to −90.56 nmol/L; *P* < 0.001), as depicted in Figure 6C.

**FIGURE 7:**
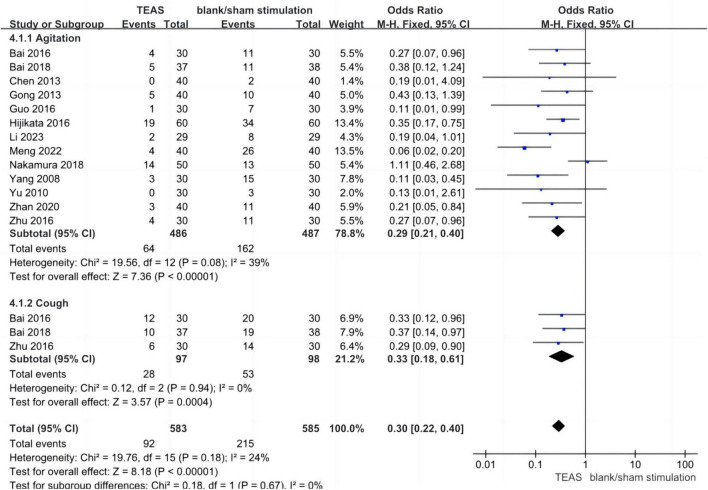
Forest plots (adverse reactions). TEAS, transcutaneous electrical acupoint stimulation group; control, blank/sham stimulation group.

Twelve studies assessed the incidence of agitation in the TEAS group versus the blank/sham stimulation group during the awakening period, encompassing 973 patients—486 in the TEAS group and 487 in the control group. The analysis did not reveal significant statistical heterogeneity (*P* = 0.08, *I*^2^ = 39%), indicating low sensitivity and good stability. A fixed-effects model was used for effect size calculation and analysis. The aggregated results indicated a significantly lower incidence of agitation in the TEAS group compared to the blank/sham stimulation group (OR, 0.29; 95% CI, 0.21–0.40; *P* < 0.001). Three studies evaluated the incidence of cough in the TEAS group versus the blank/sham stimulation group during the awakening period, involving 195 patients—97 in the TEAS group and 98 in the control group. The analysis did not reveal significant statistical heterogeneity (*P* = 0.94, *I*^2^ = 0%), indicating low sensitivity and good stability. A fixed-effects model was used for effect size calculation and analysis. The results showed a significantly lower incidence of cough in the TEAS group compared to the blank/sham stimulation group (OR, 0.33; 95% CI, 0.18–0.61; *P* < 0.001). The combined subgroup analysis demonstrated a significantly lower overall incidence of adverse reactions in the TEAS group compared to the blank/sham stimulation group (OR, 0.30; 95% CI, 0.22–0.40; *P* < 0.001), as shown in Figure 7.

The summary of the results is presented in Table 4.

**TABLE 4:** Summary of results.

Outcomes	Type	Effect sizes	Unit
Time to open eyes^※^	MD	−3.16 [−3.93, −2.39]*	min
Time to extubation^※^	MD	−4.28 [−4.79, −3.76]*	min
Time to leave the PACU^※^	MD	−8.04 [−9.48, −6.61]*	min
MAP at extubation^※^	MD	−9.00 [−10.69, −7.32]*	mmHg
HR at extubation^※^	MD	−7.62 [−9.02, −6.22]*	beats/min
Plasma epinephrine at extubation^※^	SMD	−0.81 [−1.04, −0.58]*	–
Plasma norepinephrine at extubation^※^	MD	−47.67 [−62.88, −32.46]*	pg/ml
Plasma cortisol at extubation^※^	MD	−110.92 [−131.28, −90.56]*	nmol/L
Adverse reactions^※^	OR	0.30 [0.22, 0.40]*	–

^※^There was no statistical heterogeneity.

*P-value < 0.001.

### 3.5 Publication bias

**FIGURE 8:**
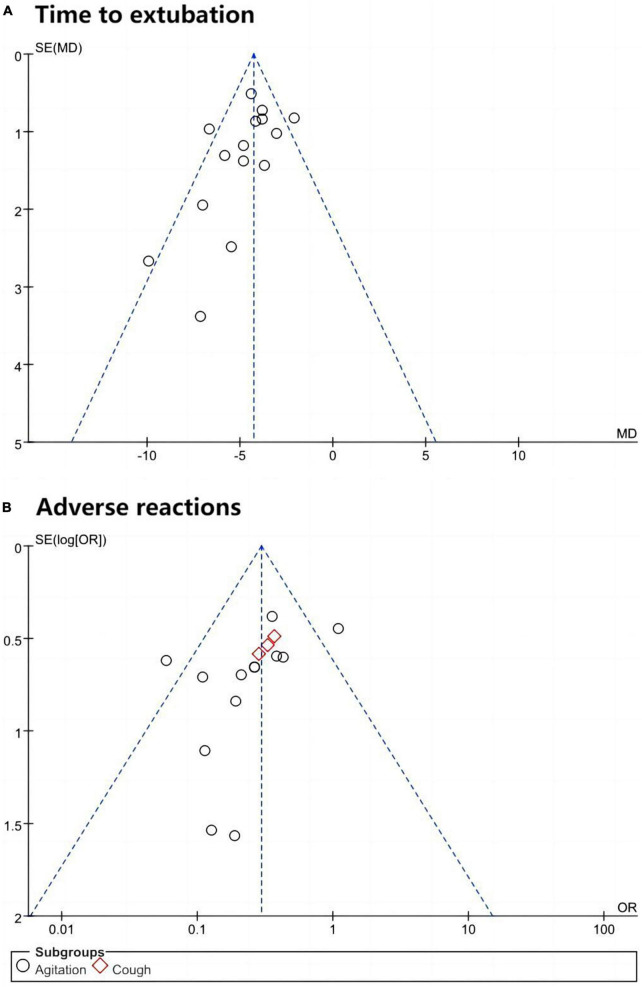
The funnel plot. **(A)** Time to extubation. **(B)** Adverse reactions.

Using “Time to extubation” and “Adverse reactions” as examples, funnel plots were constructed using Review Manager 5.3, as displayed in Figure 8. The plots exhibited poor symmetry about the central axis, and the Egger regression test results indicated significant statistical publication bias (Time to extubation: *P* = 0.066; Adverse reactions: *P* = 0.092). Furthermore, the findings of this meta-analysis should be interpreted with caution due to the potential for bias related to specific surgery types, articles from the same research team, and geographical bias.

## 4 Discussion

### 4.1 Analysis of study results

Existing meta-analyses on the perioperative applications of acupuncture therapy and acupoint stimulation encompass a diverse array of interventions. These include invasive acupuncture modalities (e.g., manual acupuncture, electroacupuncture, etc.), transcutaneous acupoint electrical stimulation, auricular point pressure beans, acupressure, and the application or implantation of threads at acupoints. This heterogeneity inevitably introduces various biases and clinical heterogeneities, which can undermine the confidence in the findings (40). To mitigate these issues, the present study exclusively included trials utilising TEAS to ensure a uniformity of intervention. The selection of acupuncture points is pivotal for the efficacy of TEAS, with the studies reviewed selecting points such as PC6, LI4, and ST36, among others. These points adhere to the established selection criteria for acupuncture and acupoint stimulation therapies during the perioperative period in recent years (3). According to contemporary principles of traditional Chinese medicine, stimulation of the acupoints PC6, LI4, and ST36 is believed to enhance the circulation of qi and blood, invigorate meridians and collaterals, alleviate pain by unblocking qi, and tranquillise the mind (12). Furthermore, the temporal parameters, frequency, and intensity of the stimulation are known to significantly influence the efficacy of TEAS. Evidence suggests that TEAS administered 30 min prior to anaesthetic induction and sustained for a minimum of 30 min can augment the effects of sedation and analgesia (17). In the majority of studies included in this analysis, the stimulation was initiated no later than 20–30 min before the commencement of anaesthesia and continued until the conclusion of the surgical procedure. The selection of electroacupuncture frequency can differentially activate neurotransmitters within the brain. For instance, stimulation at 2 Hz has been shown to trigger the release of substantial quantities of endorphins and enkephalins in both the brain and spinal cord, whereas stimulation at 100 Hz can induce the release of significant amounts of dynorphin in the spinal cord (41). The majority of studies analysed herein employed an alternating pattern of sparse and dense waves at 2 and 100 Hz, respectively. This approach is capable of simultaneously releasing the aforementioned peptides, ensuring the therapeutic effect, prolonging the duration of action, and achieving a synergistic impact.

This meta-analysis demonstrates that patients in the TEAS group experienced a more rapid awakening process, as evidenced by shorter times to eye opening, extubation, and departure from the PACU, when compared to the control group receiving blank/sham stimulation. These findings suggest that the perioperative application of TEAS may facilitate the recovery of consciousness and the awakening of patients following general anaesthesia. Additionally, the TEAS group exhibited lower MAP, HR, and plasma levels of epinephrine, norepinephrine, and cortisol at the time of extubation, indicating that TEAS can attenuate the stress response and stabilise haemodynamics during the awakening phase post-general anaesthesia. Moreover, the incidence of adverse reactions, such as agitation and coughing, was significantly reduced in the TEAS group relative to the blank/sham stimulation group, which underscores the safety of perioperative TEAS application.

### 4.2 Clinical effects and mechanisms

Investigations have demonstrated that TEAS possesses the capacity to inhibit the transmission of peripheral nociceptive information, mitigate central sensitisation, and facilitate the secretion of endogenous analgesic mediators within the central nervous system (CNS) (24). Furthermore, TEAS can modulate corresponding receptors, thereby elevating the pain threshold and exerting an adjunctive sedative influence through the inhibition of the hypothalamic-limbic system (25). In addition to these effects, TEAS has been shown to regulate systemic levels of inflammatory markers and curtail inflammatory responses (13). It also diminishes the concentration of brain oedema-associated molecules, such as Aquaporin-4 (AQP-4) and Matrix Metalloproteinase-9 (MMP-9), as well as the brain injury marker S100-β (19). TEAS exerts a regulatory influence on the neurohumoral-endocrine system, thereby preserving human physiological homeostasis and diminishing the disruptive impact of adverse stressors, such as those induced by anaesthesia and surgical trauma, on normal bodily functions.

In summary, the adjunctive application of TEAS in the perioperative period not only serves to augment the efficacy of anaesthesia and reduce the requisite dosage of intraoperative sedatives and analgesics, but also accelerates the postoperative restoration of consciousness and the awakening process in patients. TEAS also holds significant clinical importance in the realms of maintaining haemodynamic stability, attenuating adverse stress reactions and agitation during the awakening phase, and enhancing the overall quality of postoperative recovery (2, 7).

### 4.3 Limitations and implications

This study had several limitations that may have impacted the comprehensiveness of the findings. Notably, indicators such as the recovery of autonomous ventilation and the restoration of orientation were not assessed, nor was the consumption of narcotic drugs analysed, which could limit the generalisability of the results. Furthermore, inconsistencies in the units of plasma epinephrine across the included studies may have affected the reliability of the comparative analysis. Additional postoperative recovery indicators, including postoperative pain, nausea and vomiting, and cognitive function, were not examined, potentially restricting the applicability of the findings to short-term postoperative recovery outcomes.

Variations in the initiation, duration, selection of acupuncture points, and the type of equipment, current frequency, and intensity of TEAS among the included studies could have introduced heterogeneity in the outcomes. Additionally, differences in the age, gender, and surgical procedures of the patients could also account for variations in the results. Temporal and geographical disparities among the trial centres may have generated systematic errors, while the omission of patients’ occupational and educational backgrounds could further contribute to heterogeneity. Some studies lacked explicit mention of randomisation and allocation concealment or failed to report specific methodologies, which could introduce bias. Furthermore, only a subset of studies described the implementation of blinding methods; the absence of blinding can increase the risk of measurement bias. Moreover, some studies did not report the rationale for their sample size estimation, raising concerns about potential underpowered analyses.

Clearly, there is a need for multicentre, large-sample size studies that adhere to the STRICTA and CONSORT guidelines for designing and reporting clinical trials, utilising standardised outcome measures to provide high-quality, low-bias evidence to guide clinical practice.

## 5 Conclusion

In summary, the current evidence indicates that TEAS may offer potential benefits in facilitating the recovery of consciousness, reducing the time to awakening following general anaesthesia, and improving the awakening process to be smoother and safer with a lower incidence of adverse reactions. However, caution must be exercised in interpreting these findings due to the potential for bias within the included studies. Further high-quality, low-bias research is necessary to substantiate these results.

## Data availability statement

The original contributions presented in this study are included in the article/Supplementary material, further inquiries can be directed to the corresponding author.

## Author contributions

SS: Writing – original draft, Writing – review & editing. XZ: Writing – original draft, Writing – review & editing. YM: Writing – review & editing. LX: Writing – review & editing. FW: Writing – review & editing. DZ: Writing – review & editing. FS: Writing – review & editing.
