# Supplementary material for: The effect of transcutaneous electrical acupoint stimulation on postoperative awakening after general anaesthesia: a systematic review and meta-analysis
Source: Front Med (Lausanne). 2024 Sep 23;11:1347641. doi: 10.3389/fmed.2024.1347641 (PMC11457081; doi:10.3389/fmed.2024.1347641)
Supplement: Supplementary file 1 [file Table_1.docx]

***Supplementary Material***

**Supplemental Table S1**. Preferred Reporting Items for Systematic Reviews and Meta-Analyses 2020 (PRISMA 2020) Checklists

PRISMA 2020 Main Checklist

| **Topic** | **No.** | **Item** | **Location where item is reported** |
| --- | --- | --- | --- |
| **TITLE** |  |  |  |
| **Title** | 1 | Identify the report as a systematic review. | Page 1 |
| **ABSTRACT** |  |  |  |
| **Abstract** | 2 | See the PRISMA 2020 for Abstracts checklist. |  |
| **INTRODUCTION** |  |  |  |
| **Rationale** | 3 | Describe the rationale for the review in the context of existing knowledge. | Page 2 |
| **Objectives** | 4 | Provide an explicit statement of the objective(s) or question(s) the review addresses. | Page 2 |
| **METHODS** |  |  |  |
| **Eligibility criteria** | 5 | Specify the inclusion and exclusion criteria for the review and how studies were grouped for the syntheses. | Page 3 |
| **Information sources** | 6 | Specify all databases, registers, websites, organisations, reference lists and other sources searched or consulted to identify studies. Specify the date when each source was last searched or consulted. | Page 2-3, Supplemental Table S2 |
| **Search strategy** | 7 | Present the full search strategies for all databases, registers and websites, including any filters and limits used. | Page 2-3, Supplemental Table S2 |
| **Selection process** | 8 | Specify the methods used to decide whether a study met the inclusion criteria of the review, including how many reviewers screened each record and each report retrieved, whether they worked independently, and if applicable, details of automation tools used in the process. | Page 2-5 |
| **Data collection process** | 9 | Specify the methods used to collect data from reports, including how many reviewers collected data from each report, whether they worked independently, any processes for obtaining or confirming data from study investigators, and if applicable, details of automation tools used in the process. | Page 4 |
| **Data items** | 10a | List and define all outcomes for which data were sought. Specify whether all results that were compatible with each outcome domain in each study were sought (e.g. for all measures, time points, analyses), and if not, the methods used to decide which results to collect. | Page 3 |
|  | 10b | List and define all other variables for which data were sought (e.g. participant and intervention characteristics, funding sources). Describe any assumptions made about any missing or unclear information. | Page 3 |
| **Study risk of bias assessment** | 11 | Specify the methods used to assess risk of bias in the included studies, including details of the tool(s) used, how many reviewers assessed each study and whether they worked independently, and if applicable, details of automation tools used in the process. | Page 4-6 |
| **Effect measures** | 12 | Specify for each outcome the effect measure(s) (e.g. risk ratio, mean difference) used in the synthesis or presentation of results. | Page 4-6 |
| **Synthesis methods** | 13a | Describe the processes used to decide which studies were eligible for each synthesis (e.g. tabulating the study intervention characteristics and comparing against the planned groups for each synthesis (item 5)). | Page 4-6 |
|  | 13b | Describe any methods required to prepare the data for presentation or synthesis, such as handling of missing summary statistics, or data conversions. | Page 4-6 |
|  | 13c | Describe any methods used to tabulate or visually display results of individual studies and syntheses. | Page 4-6 |
|  | 13d | Describe any methods used to synthesize results and provide a rationale for the choice(s). If meta-analysis was performed, describe the model(s), method(s) to identify the presence and extent of statistical heterogeneity, and software package(s) used. | Page 4-65 |
|  | 13e | Describe any methods used to explore possible causes of heterogeneity among study results (e.g. subgroup analysis, meta-regression). | Page 4-6 |
|  | 13f | Describe any sensitivity analyses conducted to assess robustness of the synthesized results. | Page 4-6 |
| **Reporting bias assessment** | 14 | Describe any methods used to assess risk of bias due to missing results in a synthesis (arising from reporting biases). | Page 4-7 |
| **Certainty assessment** | 15 | Describe any methods used to assess certainty (or confidence) in the body of evidence for an outcome. | Table 3 |
| **RESULTS** |  |  |  |
| **Study selection** | 16a | Describe the results of the search and selection process, from the number of records identified in the search to the number of studies included in the review, ideally using a flow diagram. | Figure 1 |
|  | 16b | Cite studies that might appear to meet the inclusion criteria, but which were excluded, and explain why they were excluded. | Figure 1 |
| **Study characteristics** | 17 | Cite each included study and present its characteristics. | Table 1,2 |
| **Risk of bias in studies** | 18 | Present assessments of risk of bias for each included study. | Figure 2,3 |
| **Results of individual studies** | 19 | For all outcomes, present, for each study: (a) summary statistics for each group (where appropriate) and (b) an effect estimate and its precision (e.g. confidence/credible interval), ideally using structured tables or plots. | Table 1,2 |
| **Results of syntheses** | 20a | For each synthesis, briefly summarise the characteristics and risk of bias among contributing studies. | Table 1-4 |
|  | 20b | Present results of all statistical syntheses conducted. If meta-analysis was done, present for each the summary estimate and its precision (e.g. confidence/credible interval) and measures of statistical heterogeneity. If comparing groups, describe the direction of the effect. | Page 7-9 |
|  | 20c | Present results of all investigations of possible causes of heterogeneity among study results. | Page 7-9 |
|  | 20d | Present results of all sensitivity analyses conducted to assess the robustness of the synthesized results. | Page 7-9 |
| **Reporting biases** | 21 | Present assessments of risk of bias due to missing results (arising from reporting biases) for each synthesis assessed. | Page 7-9 |
| **Certainty of evidence** | 22 | Present assessments of certainty (or confidence) in the body of evidence for each outcome assessed. | Table 3 |
| **DISCUSSION** |  |  |  |
| **Discussion** | 23a | Provide a general interpretation of the results in the context of other evidence. | Page 9-13 |
|  | 23b | Discuss any limitations of the evidence included in the review. | Page 9-13 |
|  | 23c | Discuss any limitations of the review processes used. | Page 9-13 |
|  | 23d | Discuss implications of the results for practice, policy, and future research. | Page 9-13 |
| **OTHER INFORMATION** |  |  |  |
| **Registration and protocol** | 24a | Provide registration information for the review, including register name and registration number, or state that the review was not registered. | Page 2 |
|  | 24b | Indicate where the review protocol can be accessed, or state that a protocol was not prepared. | Page 2 |
|  | 24c | Describe and explain any amendments to information provided at registration or in the protocol. | Not applicable |
| **Support** | 25 | Describe sources of financial or non-financial support for the review, and the role of the funders or sponsors in the review. | Page 13-14 |
| **Competing interests** | 26 | Declare any competing interests of review authors. | Page 14 |
| **Availability of data, code and other materials** | 27 | Report which of the following are publicly available and where they can be found: template data collection forms; data extracted from included studies; data used for all analyses; analytic code; any other materials used in the review. | Reference |

**PRIMSA Abstract Checklist**

| **Topic** | **No.** | **Item** | **Reported?** |
| --- | --- | --- | --- |
| **TITLE** |  |  |  |
| **Title** | 1 | Identify the report as a systematic review. | Yes |
| **BACKGROUND** |  |  |  |
| **Objectives** | 2 | Provide an explicit statement of the main objective(s) or question(s) the review addresses. | Yes |
| **METHODS** |  |  |  |
| **Eligibility criteria** | 3 | Specify the inclusion and exclusion criteria for the review. | Yes |
| **Information sources** | 4 | Specify the information sources (e.g. databases, registers) used to identify studies and the date when each was last searched. | Yes |
| **Risk of bias** | 5 | Specify the methods used to assess risk of bias in the included studies. | Yes |
| **Synthesis of results** | 6 | Specify the methods used to present and synthesize results. | Yes |
| **RESULTS** |  |  |  |
| **Included studies** | 7 | Give the total number of included studies and participants and summarise relevant characteristics of studies. | Yes |
| **Synthesis of results** | 8 | Present results for main outcomes, preferably indicating the number of included studies and participants for each. If meta-analysis was done, report the summary estimate and confidence/credible interval. If comparing groups, indicate the direction of the effect (i.e. which group is favoured). | Yes |
| **DISCUSSION** |  |  |  |
| **Limitations of evidence** | 9 | Provide a brief summary of the limitations of the evidence included in the review (e.g. study risk of bias, inconsistency and imprecision). | Yes |
| **Interpretation** | 10 | Provide a general interpretation of the results and important implications. | Yes |
| **OTHER** |  |  |  |
| **Funding** | 11 | Specify the primary source of funding for the review. | Yes |
| **Registration** | 12 | Provide the register name and registration number. | Yes |

*From:* Page MJ, McKenzie JE, Bossuyt PM, Boutron I, Hoffmann TC, Mulrow CD, et al. The PRISMA 2020 statement: an updated guideline for reporting systematic reviews. MetaArXiv. 2020, September 14. DOI: 10.31222/osf.io/v7gm2. For more information, visit: [www.prisma-statement.org](file:///C:\\Users\\ssk\\Downloads\\www.prisma-statement.org)

**Supplemental Table S2**. Search strategy

| **Database** | PubMed |
| --- | --- |
| **Date Searched** | 31 December 2023 |
| **Search Terms** | (acupuncture[Title/Abstract] OR electroacupuncture[Title/Abstract] OR TEAS[Title/Abstract] OR acupoint[Title/Abstract]) AND (general anesthesia[Title/Abstract] OR awakening[Title/Abstract] OR emergence[Title/Abstract] OR recovery[Title/Abstract] OR extubation[Title/Abstract] OR open eyes[Title/Abstract] OR PACU[Title/Abstract]) |
| **Results Returned** | 1642 |

| **Database** | EMBASE |
| --- | --- |
| **Date Searched** | 31 December 2023 |
| **Search Terms** | ('acupuncture'/exp OR acupuncture OR 'electroacupuncture'/exp OR electroacupuncture OR teas OR 'acupoint'/exp OR acupoint) AND ('general anesthesia'/exp OR 'general anesthesia' OR 'awakening'/exp OR awakening OR 'emergence'/exp OR emergence OR 'recovery'/exp OR recovery OR 'extubation'/exp OR extubation OR 'open eyes' OR pacu) |
| **Results Returned** | 3267 |

| **Database** | Cochrane |
| --- | --- |
| **Date Searched** | 31 December 2023 |
| **Search Terms** | (acupuncture OR electroacupuncture OR TEAS OR acupoint) AND (general anesthesia OR awakening OR emergence OR recovery OR extubation OR open eyes OR PACU) (Title/Abstract/Keyword) |
| **Results Returned** | 2291 |

| **Database** | China National Knowledge Infrastructure |
| --- | --- |
| **Date Searched** | 31 December 2023 |
| **Search Terms** | (针灸 + 针刺 + 电针 + TEAS + 穴位) AND (全身麻醉 + 唤醒 + 苏醒 + 拔管 + 睁眼 + PACU) (Title/Abstract/Keyword) |
| **Results Returned** | 1799 |

| **Database** | VIP Database |
| --- | --- |
| **Date Searched** | 31 December 2023 |
| **Search Terms** | (针灸 + 针刺 + 电针 + TEAS + 穴位) 与 (全身麻醉 + 唤醒 + 苏醒 + 拔管 + 睁眼 + PACU) (Title/ Keyword) |
| **Results Returned** | 3424 |

| **Database** | Sinomed Database |
| --- | --- |
| **Date Searched** | 31 December 2023 |
| **Search Terms** | (针灸 OR 针刺 OR 电针 OR TEAS OR 穴位) AND (全身麻醉 OR 唤醒 OR 苏醒 OR 拔管 OR 睁眼 OR PACU) (Common field) |
| **Results Returned** | 1459 |

| **Database** | WANFANG Medical |
| --- | --- |
| **Date Searched** | 31 December 2023 |
| **Search Terms** | (针灸 or 针刺 or 电针 or TEAS or 穴位) and (全身麻醉 or 唤醒 or 苏醒 or拔管 or 睁眼 or PACU) (Topic) |
| **Results Returned** | 953 |

**Supplemental Table S3**. Acupoint location

BL 2: Cuanzu (攒竹), on the face, hollow of the inner head of the eyebrow, at the supraorbital notch.

BL 10: Tianzhu (天柱), on the posterior region of the neck, transverse to the superior interspinous process of the second cervical vertebra, and in the depression of the outer margin of the trapezius muscle.

BL 59: Fuyang (跗阳), at the back of the calf, behind the outer ankle, Kunlun (BL 60) straight up 3 inches.

BL 60: Kunlun (昆仑), in the depression between the lateral malleolus and the Achilles tendon.

BL 63: Jinmen (金门), on the lateral part of the foot, directly below the anterior border of the lateral malleolus, at the inferior margin of the cuboid bone.

EX-HN 4: Yuyao (鱼腰), directly above the pupil,in the centre of the eyebrow.

GB 40: Qiuxu (丘墟), in the malleolar region, anterior and inferior to the lateral malleolus, in the lateral depression of the extensor digitorum longus tendon.

GB 20: Fengchi (风池), at the level of Fengfu (DU 16), in the depression between the upper ends of the sternocleidomastoid trapezius muscles.

HT 7: Shenmen (神门), on the palmar ulnar end of the transverse crease of the wrist,and on the radial aspect of the tendon of the ulnar flexor m. of the wrist.

LI 4: Hegu (合谷), on the dorsum of the hand, between the first and second metacarpal bones, approximately in the middle of the second metacarpal bone on the radial side, line

up the position the transverse crease of the first joint of the thumb with the margin of the web between the thumb and the index finger of the other hand.

LI 11: Quchi (曲池), at the midpoint of the line between the radial end of the cubital crease and the external humeral epicondyle.

LI 18: Futu (扶突), in the lateral part of the neck, beside the larynx, between the anterior and posterior margins of the sternocleidomastoid muscle.

LU 7: Lieque (列缺), on the radial aspect of the forearm, between the tendons of the abductor pollicis longus and the extensor pollicis brevis muscles, in the groove for the abductor pollicis longus tendon, 1.5 *cun* superior to the palmar wrist crease.

LU 5: Chize (尺泽), on the cubital crease,and the radial aspect of the tedon of the biceps m. of the arm.

LR 3: Taichong (太冲), in the depression anterior to the junction of 1st and 2nd metatarsal bones.

SJ 5 (TE 5): Waiguan (外关), on the line joining Yangchi (SJ 4) and the tip of elbow, 2 *cun* above the dorsocarpal transverse crease, between the ulna and radius.

ST 9: Renying (人迎), In the neck, 1.5 *cun* next to the Adam's Apple, the leading edge of the sternocleidomastoid, the pulse of the common carotid artery.

ST 36: Zusanli (足三里), 3 *cun* below Dubi (ST 35), one finger breadth from the anterior crest of the tibia.

SP 6: Sanyinjiao (三阴交), on the medial side of the leg, 3 *cun* above the tip of the medial malleolus, posterior to the medial border of the tibia.

PC 6 (P 6): Neiguan (内关), on the palmar side of the forearm and on the line connecting Quze (PC3) and Daling (PC7), 2 *cun* above the crease of the wrist.

PC 8: Laogong (劳宫), on the radial aspect of the 3rd metacarpal bones, at the tip of the middle finger when make a fist and the finger is flexed.

RN 4: Guanyuan (关元), on the anterior midline, 3 *cun* below the umbilicus.

RN 3: Zhongji (中极) , on the anterior midline, 4 *cun* below the umbilicus.
